# A graphene hybrid supramolecular hydrogel with high stretchability, self-healable and photothermally responsive properties for wound healing†

**DOI:** 10.1039/d0ra09106e

**Published:** 2021-02-04

**Authors:** Haifeng Zhang, Shiya Zheng, Canwen Chen, Dagan Zhang

**Affiliations:** Department of Surgery, Nanjing Center Hospital Nanjing 210000 China; Zhongda Hospital, School of Medicine, Southeast University Nanjing 210009 China; Department of General Surgery, Affiliated Jinling Hospital, Medical School of Nanjing University Nanjing 210002 China njuccw@qq.com; Institute of Translational Medicine, The Affiliated Drum Tower Hospital, Medical School of Nanjing University Nanjing 210008 China

## Abstract

Wound healing is a ubiquitous healthcare problem in clinical wound management. In this paper, the fabrication of a graphene hybrid supramolecular hydrogel (GS hydrogel) for wound dressing applications is demonstrated. The hydrogel is composed of two components, including *N*-acryloyl glycinamide (NAGA) as the scaffold and graphene as the photothermally responsive active site for photothermal therapy. Based on the multiple hydrogen bonds between the dual amide motifs in the side chain of *N*-acryloyl glycinamide, the hydrogel exhibits high tensile strength (≈1.7 MPa), good stretchability (≈400%) and self-recoverability. In addition, the GS hydrogel shows excellent antibacterial activity towards methicillin-resistant *Staphylococcus aureus* (MRSA), benefiting from the addition of graphene that possesses great photothermal transition activity (≈85%). Significantly, *in vivo* animal experiments also demonstrated that the GS hydrogel effectively accelerates the wound healing processes by eradicating microbes, promoting collagen deposition and angiogenesis. In summary, this GS hydrogel demonstrates excellent mechanical performance, photothermal antimicrobial activity, and promotes skin tissue regeneration, and so has great application potential as a promising wound dressing material in clinical use.

## Abstract 2

The hydrogel demonstrated properties with high stretchability, self-healable and photothermal properties. Notably, photothermal therapy could be established due to its photothermal responsiveness, benefiting infected wound healing.

## Graphical abstract

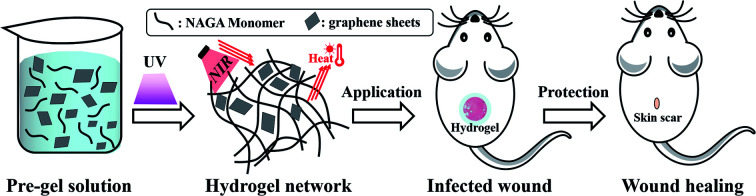

## Introduction

Skin, as the most exposed epithelial tissue, lines the body, providing an initial protective barrier, but it is also vulnerable.^1–5^ Following damage or a trauma, complex biological processes, including interactive and overlapping phases: hemostasis, inflammation, proliferation, and remodeling/maturation, occur due to the loss of inherent skin protection.^6–8^ Furthermore, once the wounds become infected, cutaneous injuries are painful and problematic, leading to high treatment costs and causing negative effects on individual patients, their families, and societies.^9–13^ In order to solve this problem, many wound dressing products, such as gauzes, nanofibers, sponges, foams, and hydrogels, amongst others, have been fabricated to promote wound healing.^14–17^ Among these products, hydrogels have emerged as a promising option, because they inherit a three-dimensional (3D) porous network, provide a moist environment that mimics the extracellular matrix (ECM) and can, therefore, facilitate the exchange of oxygen and nutrients, and bring about epidermal cell migration.^18–22^ However, although these hydrogels offer many useful properties, irreversible crosslinking always results in a hydrogel that is unable to heal after breaking, making such hydrogels ineffective in maintaining their function during use. Thus, a beneficial hydrogel with a self-healing property that can play a long-term protective role in wound healing is still highly anticipated.

In this paper, a near-infrared (NIR) light-responsive graphene hybrid supramolecular hydrogel with high stretchability, self-healable and photothermally responsive properties, was developed for wound healing. It is worth noting that the emergence of self-healing supramolecular hydrogels has injected new energy into the field of tissue engineering including wound healing, due to advantages such as tissue mimicry, biocompatibility, and injectability, which are endowed by the reversibly cross-linked polymer network of the hydrogel.^23–27^*N*-Acryloyl glycinamide, whose systematic name is *N*-(carbamoylmethyl)prop-2-enamide, C_5_H_8_N_2_O_2_, possesses a dual amide that can form multiple stable hydrogen bonding domains, which can serve as physical cross-linkages for the supramolecular polymer hydrogel.^28,29^ In addition, the tissue-specific microenvironment, such as inflammation, is an important design component of effective wound dressings.^30–34^ Thus, effective antibacterial treatments are necessary. Recently, graphene and its derivatives, as emerging two-dimensional (2D) materials, have been studied widely in many fields, such as in biomedical science, energy, and environmental applications, because of their advantages of electrical, mechanical, and thermal properties.^35–38^ It should be noted that much less efforts have been made in the preparation of graphene hybrid supramolecular hydrogels. Therefore, the development of more graphene composited supramolecular hydrogels is urgently required, further promoting their biomedical applications, such as for wound healing.

Herein, a graphene hybrid supramolecular hydrogel was generated by combining graphene with a NAGA hydrogel. It was found that the solo NAGA hydrogel exhibited good stretchability and self-healing. In addition, the graphene hybrid hydrogel preserved its good elasticity in a decreasing trend with increasing concentration of graphene in the composited hydrogel, but this hardly affected its self-healing performance. Furthermore, the hybrid hydrogel was endowed with good NIR-responsive photothermal activity by the introduction of graphene. Clearly, upon the addition of graphene, the magnitude of the photothermal response of the GS hydrogel could be significantly increased. Meanwhile, the GS hydrogel shows excellent antibacterial activity against MRSA, benefiting from the great photothermal transition activity of the graphene. Significantly, *in vivo* animal experiments also demonstrated that the GS hydrogel effectively accelerates the wound healing processes by eradicating microbes and promoting collagen deposition and angiogenesis. These features indicate that our GS hydrogels have great potential for clinically translated tissue engineering therapies.

## Materials and methods

### Materials

*N*-Acryloyl glycinamide, nano graphene sheets and 2-hydroxy-4′-(2-hydroxyethoxy)-2-methylpropiophenone were all purchased from Sigma-Aldrich (USA) and were used as received. The antibodies CD31, TNF-α, and α-smooth muscle actin (α-SMA) were obtained from Nanjing Microworld Biotechnology Co., Ltd (China). The male Sprague Dawley rats (8–12 weeks) were supplied by the Department of Comparative Medicine of Jinling Hospital (Nanjing, China). All animals were treated in strict accordance with the recommendations in the Guide for the Care and Use of Laboratory Animals of the National Institutes of Health, USA. All experimental protocols and care of the animals were reviewed and approved by the animal research committee of the Jinling Hospital.

### Synthesis and characterization of GS hydrogels

PNAGA hydrogels were prepared using the following procedure. First, the NAGA monomer was dissolved in deionized water. Then, 1 wt% of the photo-initiator 2-hydroxy-4′-(2-hydroxyethoxy)-2-methylpropiophenone (2959) was added to the monomer solution and vortexed vigorously at 50 °C. Subsequently, 1–1.5 ml of the solution was injected into a plastic cylinder mold. Photopolymerization was performed for 10 min at room temperature in a sealed ultraviolet light irradiated environment (365 nm, 100 W). During this process, the graphene hybrid hydrogel was generated by integrating nanographene sheets at different concentrations. The resulting graphene hybrid supramolecular hydrogels were named GS hydrogels and were labeled according to the concentration of nanographene sheets. For example, GS (1) refers to the hydrogel with a nanographene concentration of 1%.

### Self-healability of the GS hydrogels

The resulting hydrogel bars were fully equilibrated in PBS and an oblique cut was created from the middle. Thereafter, the two separated pieces were partially and completely in contact with each other in a specific mold. The molds were immersed in a water bath at 55 °C for 45 minutes. In order to prevent the evaporation of water during heating, plastic wrap was used to tightly seal the mold. Finally, the hydrogels were removed from the molds and were tested for their self-healing efficiency. These hydrogels, the control gel and the nanocomposite hydrogels, were stretched to test the self-healing behavior by observing the unity of incisions and the stretchability.

### Photothermal performance of the GS hydrogels

GS hydrogels containing different concentrations of nanographene sheets were irradiated with an 808 nm laser (2 W cm^−2^) for 5 seconds on the same white background, in order to monitor the photothermal conversion performance of the hydrogels. Thermal images were taken with an NEC Avio Thermo Tracer TH9100 thermal camera.

### Antibacterial activity of the GS hydrogels

To evaluate the antimicrobial activity of the GS hydrogels, the drug-resistant pathogen methicillin-resistant *Staphylococcus aureus* (MRSA) was used as a model strain. Bacterial suspensions were prepared according to the McFarland criteria, and MRSA was added to PBS tubes and shaken until the turbidity was 0.5. Subsequently, the bacteria suspension was inoculated onto agar plates for 48 hours and different hydrogels were placed onto the agar surface, including GS (0), GS (2.5) without NIR irradiation, and GS (2.5) subjected to NIR irradiation. After 3 days of co-culture, the zone of inhibition (ZOI) was estimated by the distance between the outer diameter of the zone of inhibition and the outer diameter of the hydrogel, hence determining the inhibition of the hydrogel.

### *In vivo* wound healing experiments

All experimental protocols used in this study constituted appropriate and acceptable procedures and were carried out according to the requirements of the previously mentioned committee. A hole approximately 1 cm in diameter was made in the back skin of the SD male mice and 100 μL of *Staphylococcus aureus* solution (10^8^ CFU ml^−1^) was dripped onto the wound to establish an infected wound model. The mice were then equally divided into three groups that received different treatment, including PBS, GS (0) and GS (2.5), respectively. Each group applied NIR (2 W cm^−2^) to the wound site for 10 minutes. Photographs of the wound sites were taken on the day of operation (op), day 3, day 5, and day 7. The wound healing ratio was calculated using the equation: [1 − (the current area of the wound)/(the initial area of the wound)] × 100%. Seven days later, the rats were sacrificed, and granulation tissues over the wound were excised for H&E staining, Masson's trichrome staining, and immunohistochemical evaluation. Sections for immunohistochemical evaluation were stained with IL-6 and TNF-α. For neovascularization evaluation, sections were reacted with primary antibodies CD31 (KEYGEN and KGYM0118-7) and α-smooth muscle actin (KEYGEN and KGYT5053-6) overnight at 4 °C.

## Results and discussion

**Fig. 1:**
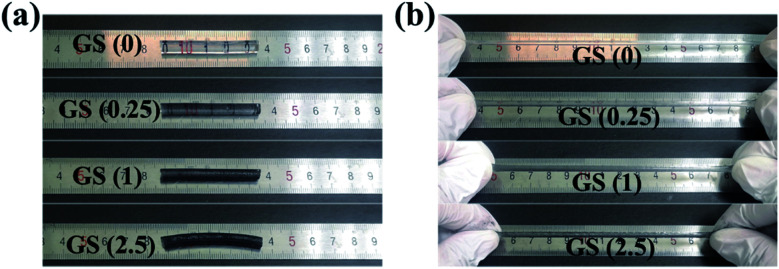
Deformation of the different GS hydrogels: (a) the original state of the GS hydrogels, (b) the state of the hydrogels after stretching.

Briefly, the fabrication of GS hydrogels comprising NAGA and graphene were prepared *via* photoinitiated copolymerization. Different GS hydrogels were prepared by integrating different graphene concentrations (0, 0.25, 1, and 2.5 mg ml^−1^) into NAGA monomer solutions. It was observed that the graphene nanosheets showed layered stacks of graphene with radial widths of several to tens of microns (Fig. S1†). Then, the hydrogels were labeled according to the concentration of graphene. For example, GS (2.5) refers to the hydrogel with a graphene concentration of 2.5 mg ml^−1^. After being generated, the stretching ability of the different GS hydrogels was first tested. As shown in Fig. 1a, four GS hydrogels, GS (0), GS (0.25), GS (1), and GS (2.5), were all made into cylinders of the same length of approximately 4.5 centimeters. In addition, GS (5) was also developed, however it was observed that, in this case, polymerization of the gel did not occur, even after a long time of 12 hours of UV light irradiation, as shown in Fig. S2.† It is possible that the deep black of the high graphene concentration blocked the UV light-initiated gel polymerization reaction (Fig. S2†). Furthermore, it was observed that the GS hydrogels exhibited good elasticity (Fig. 1b). Among the hydrogels, the largest stretchability was found in the GS (0) group (≈400%), followed by the GS (0.25) group (≈350%), then the GS (1) group (≈300%), and the smallest stretchability was in the GS (2.5) group (≈250%). The tensile strengths of the different hydrogels were examined and are depicted as stress *vs.* strain curves, as shown in Fig. S3.† It was found that the mechanical properties were influenced by the graphene content. At a fixed NAGA monomer concentration, increasing the graphene content led to the amelioration of mechanical properties. In addition, as the proportion of graphene in the GS hydrogel increased, the stretchable properties of the hydrogel gradually weakened. The reason for the reduced stretchability is that graphene, as a nanoscale material, can fill the voids of the hydrogel networks, increasing the strength of the hydrogel, but reducing the flexibility of the hydrogel. Next, other physical properties of the GS hydrogels, such as drying and re-swelling, were investigated (Fig. S4†). The re-swelling performance of the GS hydrogels was evaluated by drying the samples in air and then dipping them into DI water to swell. It was clear that the GS hydrogels with different graphene content all exhibited excellent and similar re-swelling performance, suggesting that the addition of more graphene did not significantly affect this physical property of the hydrogels.

**Fig. 2:**
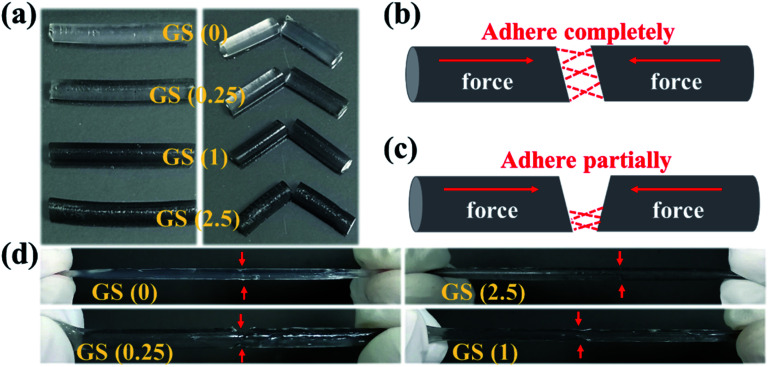
Self-healing performance of the different GS hydrogels: (a) hydrogels and their corresponding fragments cut with a scalpel; the schematic illustration of the two parts of the hydrogels healed completely (b) and partially (c); images of the healed hydrogels (d).

Furthermore, in order to detect the influence of the introduction of graphene on the self-healing ability of the GS hydrogel, the four GS hydrogels also underwent self-healing experiments (Fig. 2). Briefly, the fabricated four cylindrical hydrogels were respectively cut into two segments (Fig. 2a). Subsequently, the two segments in each group were assembled in a complete or partial way (Fig. 2b or Fig. 2c). As shown in Fig. 2d, groups GS (0) and GS (2.5), which assembled in a complete way, displayed an excellent self-repairing effect, forming an integrated cylindrical gel in the absence of traces of cutting. In addition, for the other groups that healed in a partial approach, excellent healing was also observed where the two segments touched. These results indicate that GS hydrogels inherit excellent self-repairing properties, brought about by hydrogen bonds in NAGA that has high recovery and reversibility. In addition, the self-healing hydrogels show elasticity that is as good as their original elasticity. We can attribute the great self-healing capability to the dissociation and reformation of hydrogen bonds at the pre-broken interface.

**Fig. 3:**
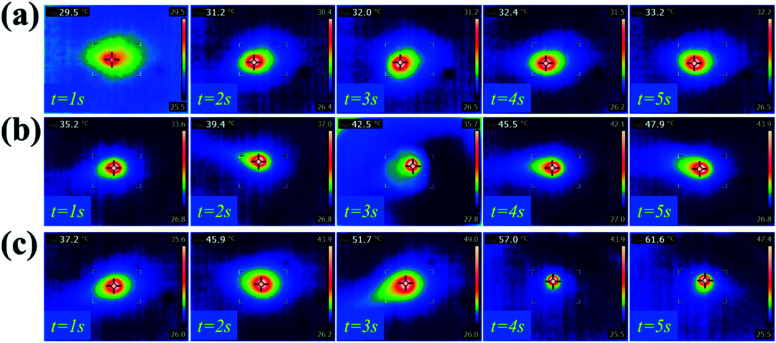
Photothermal properties of the hydrogels: infrared thermographs of the GS (0.25) hydrogel (a), the GS (1) hydrogel (b), and the GS (2.5) hydrogel (c) under NIR laser irradiation for 5 seconds.

Graphene, as a novel material that has won the Nobel Prize, possesses excellent optical, electrical and mechanical properties, such as good NIR-responsive photothermal activity. Here, GS hydrogels with different concentrations of graphene were employed to study the NIR photothermal performance of graphene hybrid hydrogels. As shown in Fig. 3, the photothermal capacity of the GS hydrogels were measured by irradiating each sample with an NIR laser for 5 s. The different GS hydrogels, GS (0.25), GS (1), and GS (2.5), were placed in the same environment at room temperature (25 °C). After 5 seconds of NIR irradiation, the maximum temperature of 61.6 °C was observed in the GS (2.5) hydrogel, followed by a temperature of 47.9 °C in the GS (1) hydrogel, and then 33.2 °C in the GS (0.25) hydrogel (Fig. 3a–c). The above results demonstrate that the maximum attainable temperature could be significantly improved by increasing the relative mass of graphene in the hydrogel (Fig. 3d). In particular, the temperature of the GS (0.25) hydrogel increased by 10 °C after 5 s of NIR irradiation. For the GS (1) specimen, a 2.5 times increase of the temperature could be obtained, and for the GS (2.5) hydrogel, an almost 40 °C increase could be obtained following irradiation for 5 s. As reported, the graphene also showed highly repeatable photothermal properties. The GS (2.5) hydrogel, as a representative example, was further employed to confirm the photothermal characteristics (Fig. S5†). After four cycles of NIR irradiation and free cooling, the hydrogels still maintained their NIR photothermal responsive properties, indicating that the hydrogels were capable of excellent photothermal transition capacities, as shown in Fig. S6.†

Notably, photothermal therapy is recognized as a superb antibacterial method without any risk of inducing drug resistant behavior, and this is related to the physical damage of the bacteria by heating above 50 °C. Here, MRSA bacteria, which is the most common pathogenic drug-resistant bacteria in clinical practice, was chosen to evaluate the antimicrobial properties of the hydrogels with and without NIR irradiation. As presented in Fig. S7a,† the GS (0) scaffolds showed limited antibacterial activity even with NIR irradiation. However, although graphene has been widely used to inhibit the growth of bacteria, the antibacterial effect was not prominent without NIR illumination (Fig. S7b†). It is possible that the direct contact of graphene with the bacteria was hindered by the hydrogel scaffold, thus weakening its antibacterial effect. Conversely, once irradiated by NIR for 5 s with four cycles was carried out, the GS (2.5) hydrogel presented good antibacterial behavior and showed a clear ZOI, proving that the heat caused by the hydrogel after irradiation will dissipate and result in the reduction of the bacterial population (Fig. S7c†). The possible reason for this phenomenon is an elevated local temperature by the photosensitizer graphene, leading to denaturation of microbial proteins and further leading to the death of pathogenic microorganisms.^38^ Its antibacterial effect was also observed by live-dead fluorescent staining of MRSA, as shown in Fig. S7d–g.† These results demonstrate the significant antibacterial ability of GS (2.5) with NIR illumination, compared with the other groups.

**Fig. 4:**
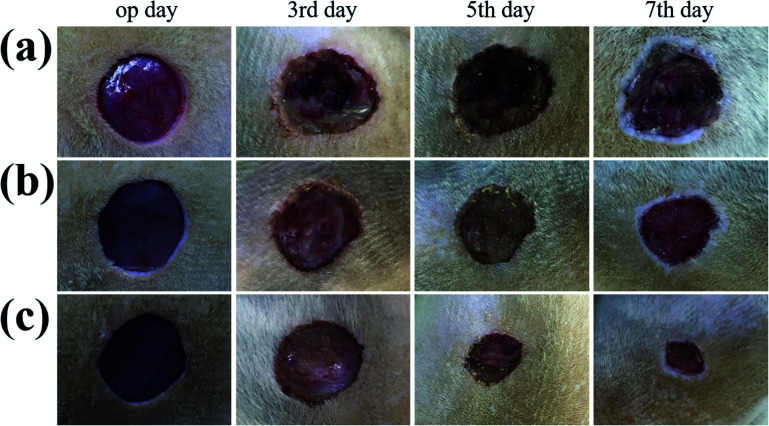
*In vivo* assessment of the GS hydrogels for wound healing. (a–c) Representative photographs of the skin wounds treated with PBS, GS (0), and GS (2.5) on day 0, day 3, day 5, and day 7. The scale bar is 500 mm.

**Fig. 5:**
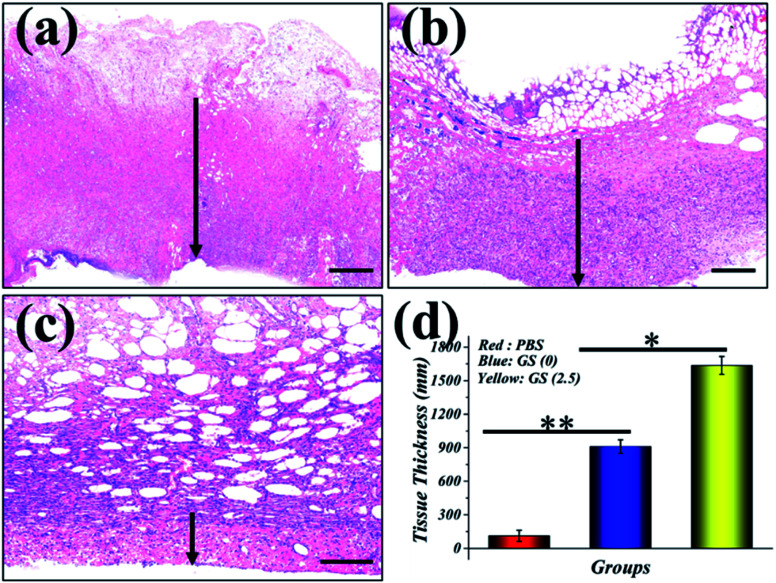
Representative H&E-stained histologic longitudinal sections of the different groups: (a) GS (2.5) hydrogel; (b) GS (0) hydrogel; (c) PBS. (d) Statistical analysis of the relative connective tissue thickness in the three groups. Data are presented as a mean with standard deviation as error bars, where *P < 0.05 and **P < 0.01. The scale bars are 500 μm in (a), 250 μm in (b), and 100 μm in (c).

In order to evaluate the effectiveness of the GS hydrogel for wound healing, a *Staphylococcus aureus* (*S. aureus*) bacterium infected full-thickness skin defect model was established on the back of SD rats. After modeling, different interventions, including phosphate buffer solution (PBS), GS (0) hydrogel, and GS (2.5) hydrogel, were employed to treat the infected wounds on these rodents, and each group received the same NIR irradiation. During the experiment, the rats all displayed free movements and normal behavior and were sensitive to sound, light, and other stimulations. As demonstrated in Fig. 4 and in Fig. S8,† the control PBS group experienced the worst wound healing with clear infection. Secondly, the GS (0) group had relatively better wound healing, but it was not as good as the GS (2.5) group, which had the best wound closure. The wound closure results show that the GS hydrogel combined with clinical photothermal physiotherapy is beneficial to improving wound tissue regeneration. Moreover, the quality of wound repair will affect the systematic states of these animals. It can be inferred that there is significant correlation between the repair of the wound and the weight of the corresponding mice (Fig. S9†). In the PBS group, it was found that the infected wound caused a negative prognosis in the mice, causing weight loss. In contrast, the weight of the mice not only did not decrease, but increased significantly in the GS (2.5) group, which experienced the best wound repair. In addition, compared to the above-mentioned two groups, the GS (0) group was at a moderate level. In order to explore the wound recovery in each group, HE of the tissue in the wound sites of each group was performed (Fig. 5). Clearly, the thickest granulation tissue was observed in the GS (2.5) group, followed by the GS (0) group, and was the worst in the PBS group. These results are consistent with the results of wound closure, providing strong evidence that a graphene hybrid supramolecular hydrogel combined with photothermal therapy can effectively promote wound repair.

**Fig. 6:**
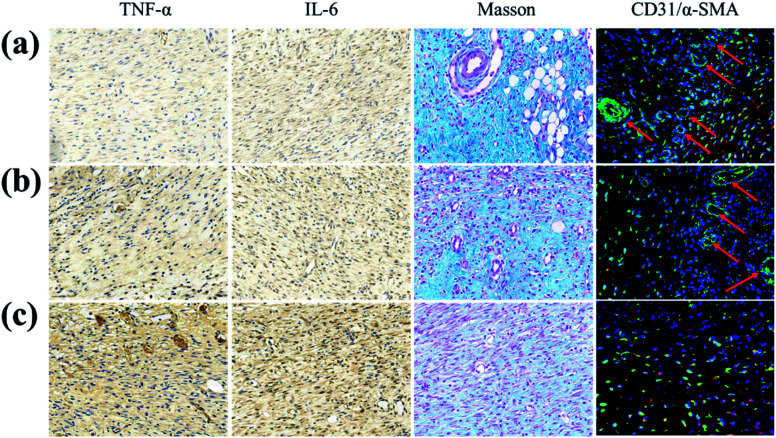
Histological and immunological analyses of the wound sections on the 7th day (a) for the GS (2.5) hydrogel, (b) for the GS (0) hydrogel, and (c) for PBS, from immunohistochemistry of TNF-α and IL-6, Masson's trichrome staining, and the double staining of CD31 and α-SMA.

Furthermore, in order to study the potential biological mechanism of a GS hydrogel in accelerating wound healing, the inflammation, collagen deposition and angiogenesis at the wound site were analyzed (Fig. 6). It was found that the pro-inflammatory factors (TNF and IL-6) in the PBS group were significantly more up-regulated than in the other two groups. In addition, compared to the GS (0) group, TNF and IL-6 in the GS (2.5) group were significantly down-regulated, reflecting its positive role in alleviating inflammation, caused by an excellent photothermal responsive performance. Similarly, compared with the other groups, GS (2.5) had the most sufficient collagen deposition, suggesting the best skin tissue regeneration. More importantly, angiogenesis is essential for tissue regeneration and remodeling. Interestingly, the GS (2.5) group developed mature vascular networks that are good in aiding wound healing. However, these vascular structures were absent in the PBS group, leading to poor tissue healing. Compared with the control group, the GS (0) group possessed certain blood vessel formation but was far inferior to the GS (2.5) group, revealing that photothermal therapy based on a graphene composite supramolecular hydrogel can promote vascular regeneration (Fig. S10†). This also highlighted the fact that our hydrogel with high stretchability, self-healable and photothermally responsive properties could play a pivotal role in tissue engineering.

## Conclusion

In summary, the prepared hydrogels demonstrated good self-healable, photothermal and wound healing promoting properties. The hydrogel is composed of two components, including a NAGA hydrogel scaffold and photothermally responsive active graphene. It was demonstrated that the GS hydrogel could self-heal after damage, due to the multiple hydrogen bonds in the hydrogel networks. In addition, it presented good antibacterial behavior against MRSA, and this depends on photothermal therapy because of its photothermic responsive ability. In an acute wound infection model, the GS hydrogel significantly promoted granulation-tissue formation by alleviating inflammation, improving angiogenesis and collagen deposition, indicating that our developed hydrogel has broad potential in wound healing as a biomimetic substrate.

## Conflicts of interest

The authors declare no conflicts of interest.

## Supplementary Material

RA-011-D0RA09106E-s001
