# Correction: A graphene hybrid supramolecular hydrogel with high stretchability, self-healable and photothermally responsive properties for wound healing

**DOI:** 10.1039/d2ra90001g

**Published:** 2022-01-18

**Authors:** Haifeng Zhang, Shiya Zheng, Canwen Chen, Dagan Zhang

**Affiliations:** Department of Surgery, Nanjing Center Hospital Nanjing 210000 China; Zhongda Hospital, School of Medicine, Southeast University Nanjing 210009 China; Department of General Surgery, Affiliated Jinling Hospital, Medical School of Nanjing University Nanjing 210002 China njuccw@qq.com; Institute of Translational Medicine, The Affiliated Drum Tower Hospital, Medical School of Nanjing University Nanjing 210008 China

## Abstract

Correction for ‘A graphene hybrid supramolecular hydrogel with high stretchability, self-healable and photothermally responsive properties for wound healing’ by Haifeng Zhang *et al.*, *RSC Adv.*, 2021, **11**, 6367–6373, DOI: 10.1039/d0ra09106e.

The authors regret that an incorrect version of Fig. 6 was included in the original article. The correct version of Fig. 6 is presented below.

**Fig. 6:**
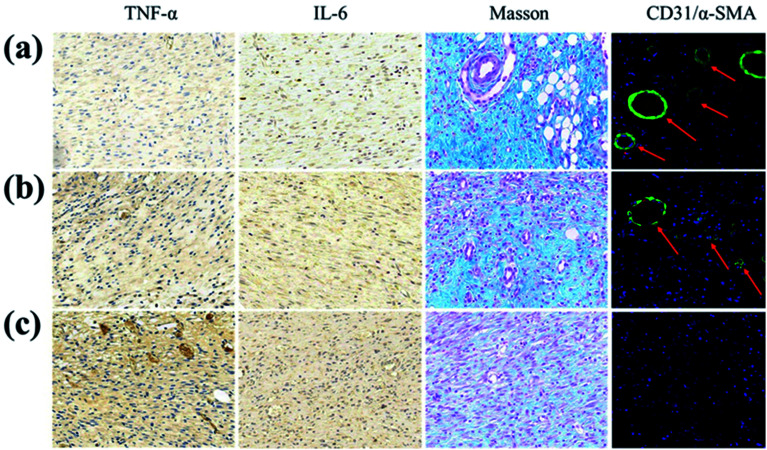
Histological and immunological analyses of the wound sections on the 7th day (a) for the GS (2.5) hydrogel, (b) for the GS (0) hydrogel, and (c) for PBS, from immunohistochemistry of TNF-α and IL-6, Masson’s trichrome staining, and the double staining of CD31 and α-SMA.

The Royal Society of Chemistry apologises for these errors and any consequent inconvenience to authors and readers.

## Supplementary Material

