# Supplementary material for: A graphene hybrid supramolecular hydrogel with high stretchability, self-healable and photothermally responsive properties for wound healing
Source: RSC Adv. 2021 Feb 4;11(11):6367–73. doi: 10.1039/d0ra09106e (PMC8694836; doi:10.1039/d0ra09106e)
Supplement: RA-011-D0RA09106E-s001 [file RA-011-D0RA09106E-s001.pdf]

Electronic Supplementary Material (ESI) for RSC Advances.
This journal is © The Royal Society of Chemistry 2021

Electronic Supporting Information

A graphene hybrid supramolecular hydrogel with high stretchable, self-healable and photothermally responsive for wound healing

Haifeng Zhang, Shiya Zheng*, Canwen Chen*, Dagan Zhan*

Department of Surgery, Nanjing Center Hospital, Nanjing 210000, China

Department of General Surgery, Affiliated Jinling Hospital, Medical School of Nanjing University, Nanjing 210002, China

Guangdong Key Laboratory of Biomedical Measurements and Ultrasound Imaging, Department of Biomedical Engineering, Shenzhen University, Shenzhen 518060, China

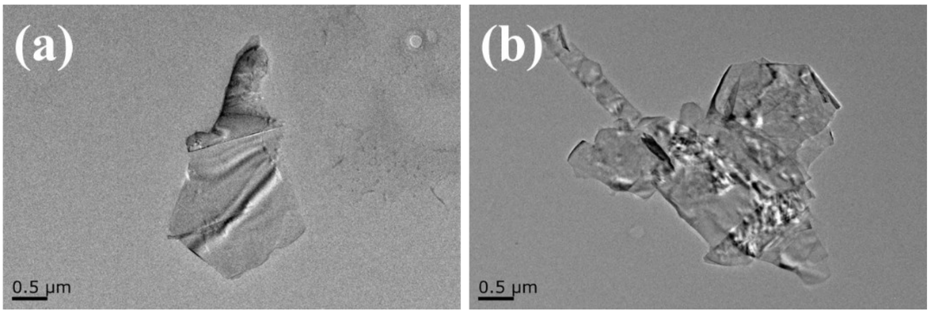

Figure S1. The TEM of graphene nanosheets.

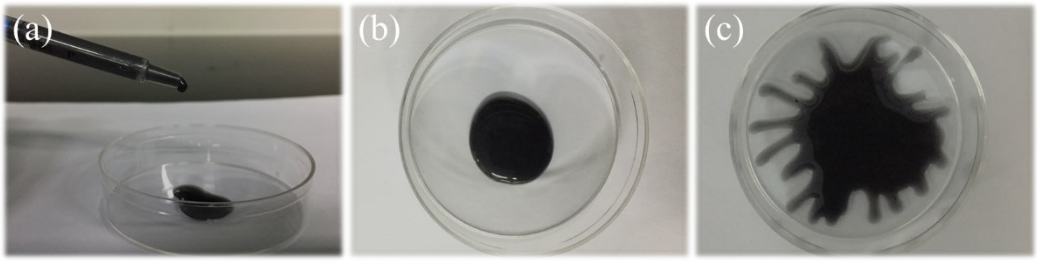

Figure S2. The images of unpolymerized GS (5). The GS (5) injectable pre-gel solution after 12 hours of UV irradiation (a); the pre-gel solution in a dish before (b) and after artificial scribbles (c).

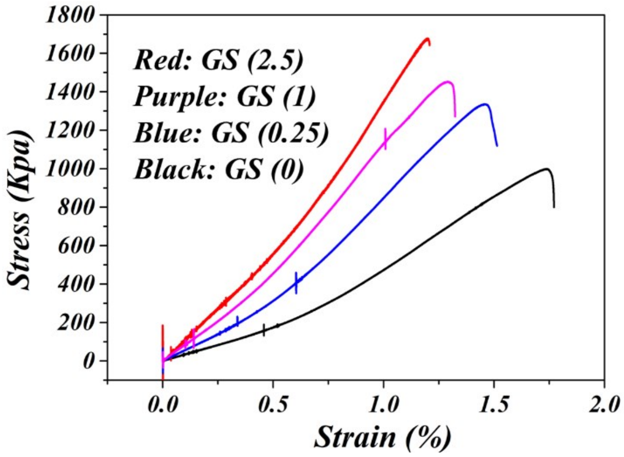

Figure S3. Tensile and stress vs strain curves of GS hydrogels.

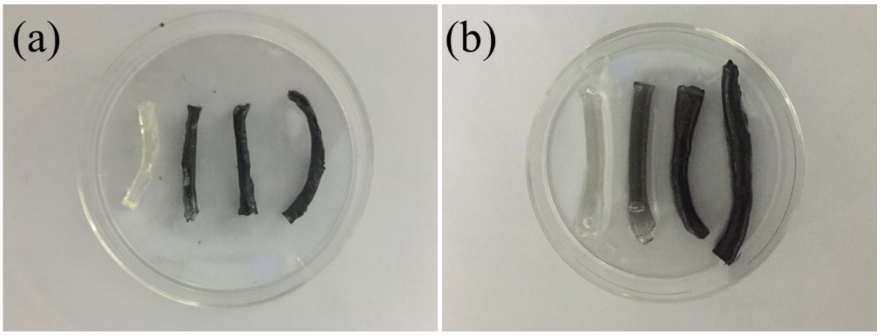

Figure S4. The images of GS hydrogels after dry and reswelling state. From left to right: GS (0), GS (0.25), GS (1), GS (2.5).

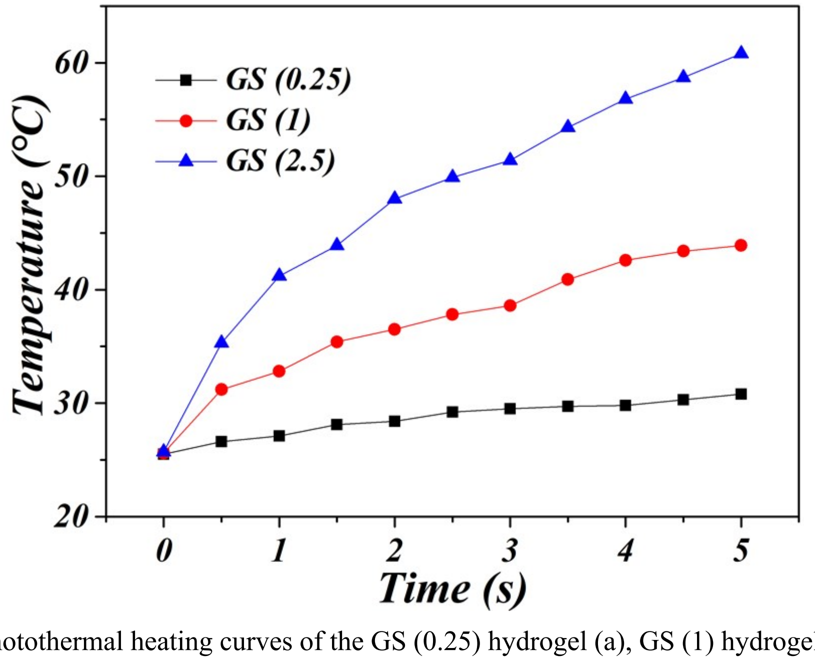

Figure S5. Photothermal heating curves of the GS (0.25) hydrogel (a), GS (1) hydrogel (b), and GS (2.5) hydrogel.

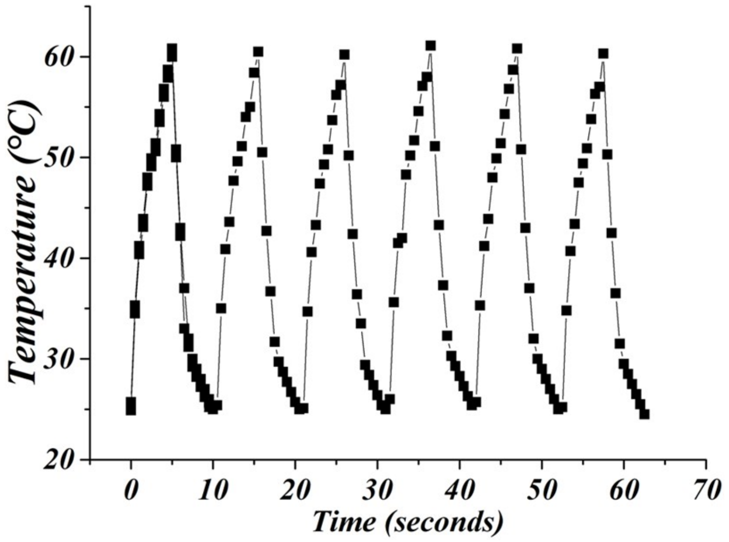

Figure S6. Temperature variation of the GS (2.5) hydrogel with repeated NIR irradiation on/off for 4 cycles.

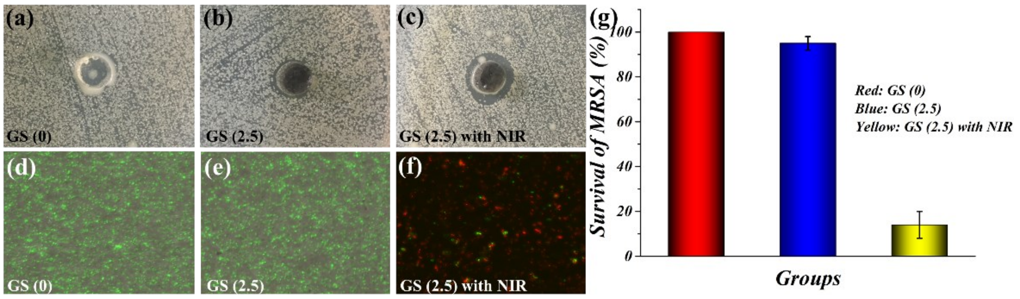

Figure S7. Antibacterial activity of GS hydrogels. Optical images of GS hydrogels the with or without NIR laser NIR laser irradiation against MRSA (a-c);

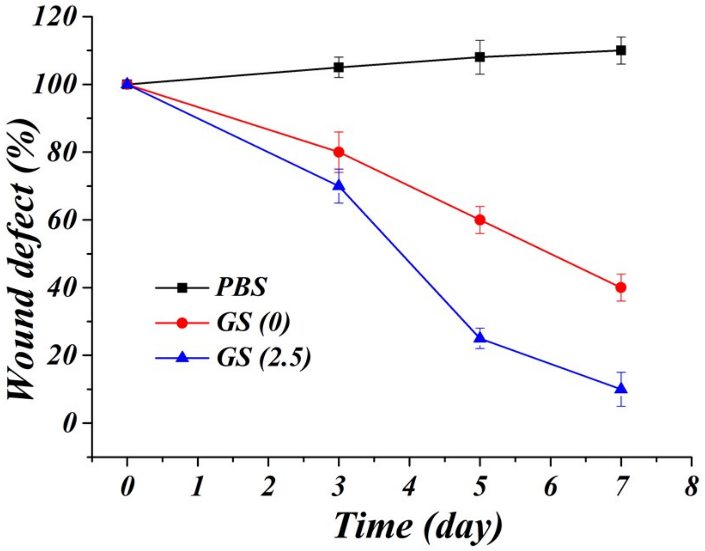

Figure S8. The changes of mean wound area in groups of PBS, GS (0), and GS (2.5) during the experiment.

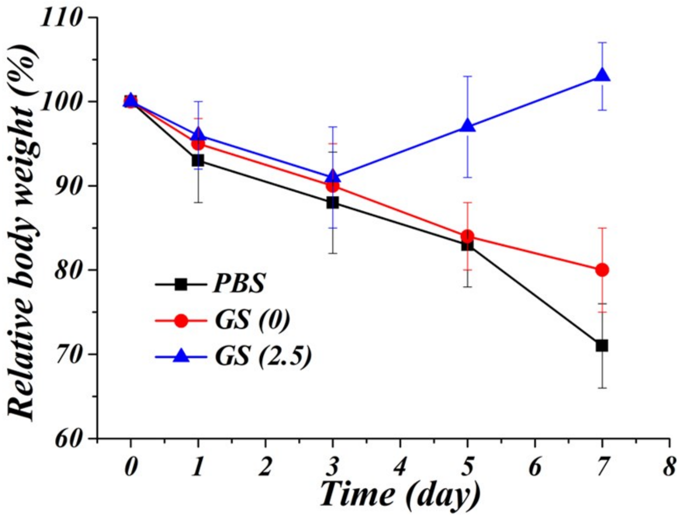

Figure S9. Quantitative analysis of the relative body weight in groups of PBS, GS (0), and GS (2.5) during the experiment.

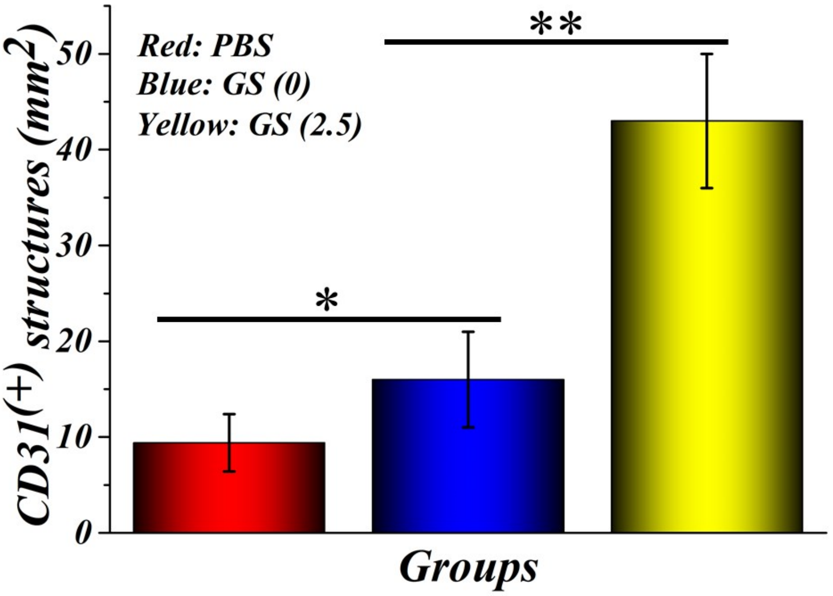

Figure S10. Quantification of the CD31 and α-SMA co-labeled structures in groups of PBS, GS (0), and GS (2.5). *P < 0.05, **P < 0.01.
